# Dysregulation of *RNF213* promotes cerebral hypoperfusion

**DOI:** 10.1038/s41598-018-22064-8

**Published:** 2018-02-26

**Authors:** Takaaki Morimoto, Jun-ichiro Enmi, Yorito Hattori, Satoshi Iguchi, Satoshi Saito, Kouji H. Harada, Hiroko Okuda, Yohei Mineharu, Yasushi Takagi, Shohab Youssefian, Hidehiro Iida, Susumu Miyamoto, Masafumi Ihara, Hatasu Kobayashi, Akio Koizumi

**Affiliations:** 1 Department of Health and Environmental Sciences, Kyoto University Graduate School of Medicine, Konoe-cho, Yoshida, Sakyo-ku, Kyoto, 606–8501 Japan; 2 Department of Neurosurgery, Kyoto University Graduate School of Medicine, 54 Shogoin Kawahara-cho, Sakyo-ku, Kyoto, 606–8507 Japan; 3 Department of Investigative Radiology, National Cerebral and Cardiovascular Center, 5–7–1 Fujishirodai, Suita, Osaka, 565–8565 Japan; 4 Department of Neurology, National Cerebral and Cardiovascular Center, 5–7–1 Fujishirodai, Suita, Osaka, 565–8565 Japan; 5 Laboratory of Molecular Biosciences, Kyoto University Graduate School of Medicine, Konoe-cho, Yoshida, Sakyo-ku, Kyoto, 606–8501 Japan; 6 Department of Biomedical Sciences, College of Life and Health Sciences, Chubu University, 1200 Matsumoto-cho, Kasugai, Aichi 487–8501 Japan

## Abstract

*RNF213* is a susceptibility gene for moyamoya disease, yet its exact functions remain unclear. To evaluate the role of *RNF213* in adaptation of cerebral blood flow (CBF) under cerebral hypoperfusion, we performed bilateral common carotid artery stenosis surgery using external microcoils on *Rnf213* knockout (KO) and vascular endothelial cell-specific *Rnf213* mutant (human p.R4810K orthologue) transgenic (EC-Tg) mice. Temporal CBF changes were measured by arterial spin-labelling magnetic resonance imaging. In the cortical area, no significant difference in CBF was found before surgery between the genotypes. Three of eight (37.5%) KO mice died after surgery but all wild-type and EC-Tg mice survived hypoperfusion. KO mice had a significantly more severe reduction in CBF on day 7 than wild-type mice (KO, 29.7% of baseline level; wild-type, 49.3%; *p* = 0.038), while CBF restoration on day 28 was significantly impaired in both KO (50.0%) and EC-Tg (56.1%) mice compared with wild-type mice (69.5%; *p* = 0.031 and 0.037, respectively). Changes in the subcortical area also showed the same tendency as the cortical area. Additionally, histological analysis demonstrated that angiogenesis was impaired in both EC-Tg and KO mice. These results are indicative of the essential role of *RNF213* in the maintenance of CBF.

## Introduction

*RNF213* (Ring Finger Protein 213)/mysterin is recognised as a major susceptibility gene for moyamoya disease (MMD), which is a progressive steno-occlusive disease of the cerebral arteries^1,2^. *RNF213* is located on chromosome 17q25.3 and encodes a 591 kDa (5207 amino acid) protein that possesses two consecutive AAA+ ATPase domains and one E3 ligase domain^2,3^. The p.R4810K (c.14429 G > A: rs112735431) founder variant of *RNF213* is found in 80% of East Asian MMD patients, with strong association^1,2^. Recently, the variant was also shown to be associated with other steno-occlusive diseases, such as intracranial arterial stenosis, pulmonary hypertension and coronary artery disease^4–7^. Consequently, *RNF213* p.R4810K is now considered to be initially associated with vascular steno-occlusive regions that then give rise to the development of moyamoya collateral vessels as a compensatory adaptation to the lowered cerebral blood flow (CBF)^5,8–10^. However, the physiological and pathological roles of *RNF213* in these steno-occlusive regions remain largely unexplored.

To establish models of MMD, *Rnf213* has been genetically modified in mice; *Rnf213* knockout (KO), transgenic (Tg) and knock-in mice have been produced by our group and by others^11–14^. However, under normal conditions, neither ablation nor the expression of *Rnf213* p.R4757K (the orthologue of human *RNF213* p.R4810K) caused any cerebrovascular changes in mice^12–15^, while an *rnf213* knockdown zebrafish model showed abnormal development of craniocervical vessels^2^. These results suggest species-specific susceptibility differences for *RNF213* dysfunction, which might be explained by compensatory pathways in mammals, and are indicative of the importance of secondary insults in addition to genetic factors in the pathogenesis of vascular disorders. This concept is consistent with the involvement of environmental factor(s) in MMD aetiology, as suggested by the low penetrance of *RNF213* p.R4810K (1/200 variant carriers) in genetic epidemiological studies of MMD^2,16^.

Several studies investigating the role of *Rnf213* in genetically modified mice under stress conditions have also been performed. Under hypoxic conditions, compensatory angiogenesis in the cerebral cortex was impaired in vascular endothelial cell-specific p.R4757K transgenic (EC-Tg) mice^13^. Reduced angiogenesis was also observed in MMD induced pluripotent stem (iPS) and *RNF213* p.R4810K-overexpressing cell models under normal condition, while *RNF213* suppression by RNAi did not inhibit angiogenesis^17^. In addition, several *RNF213* mutations, other than p.R4810K, found in non-Asian MMD patients were confirmed to result in lower angiogenesis in cell models^18^. On the other hand, in KO mice, common carotid artery ligation, which induces vascular remodelling, led to the thinning of the medial layer and inhibition of intimal hyperplasia^12^. However, none of these mouse studies revealed the typical phenotypes observed in MMD, such as stenotic lesions, moyamoya vessels and cerebral infarction^12–14^. A transient cerebral ischemic model using KO mice also showed no alteration of cerebral infarction^19^. Collectively, these studies clearly demonstrate that *RNF213* plays a role in cerebrovascular angiogenesis and remodelling, but to date an obvious MMD phenotype has not been detected in *Rnf213* genetically modified mice.

Angiogenesis and vascular remodelling are known to act as compensatory mechanisms following cerebral ischemia^20,21^. Therefore, one conjecture would be that *RNF213* may affect cerebral circulation through the two processes of angiogenesis and vascular remodelling. In this study, we evaluated the role of *RNF213* in adaptation of CBF under cerebral hypoperfusion. We employed a bilateral common carotid artery stenosis (BCAS) model, which causes prolonged cerebral hypoperfusion^22–25^. We evaluated CBF and cerebrovascular changes in KO and EC-Tg mice by magnetic resonance imaging (MRI), arterial spin-labelling (ASL) MR perfusion imaging, and histopathological analyses.

## Results

### Kaplan-Meier Survival Estimates

**Figure 1:**
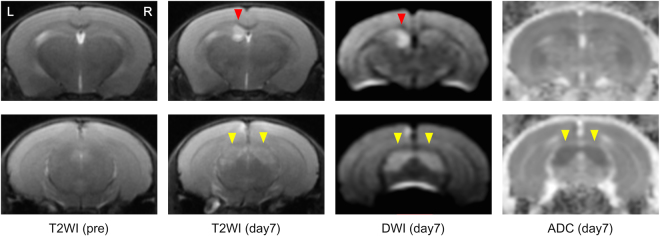
Brain magnetic resonance (MR) imaging. MR images on day 7 of the KO mouse (Animal Code: KO-5 in Supplementary Table 1) that died on day 11 after surgery are shown. Cerebral infarction is seen in the caudate nucleus (red arrowheads), and pre-Wallerian degeneration is seen in the cerebral peduncle (yellow arrowheads). T2WI, T2 weighted image; DWI, diffusion weighted image; ADC, apparent diffusion coefficient.

Three of eight KO mice were found to have died during our routine morning rounds: one died on day 1 after full recovery of BCAS surgery (day 0) and two mice died on days 8 and 11 after BCAS. In contrast, all WT and EC-Tg mice survived hypoperfusion for 28 days. One WT mouse died during anaesthesia for MRI on day 28 but, for this analysis, was regarded to have survived as it endured BCAS surgery for the predefined observation period of 28 days. Kaplan-Meier analysis showed a significantly worse survival rate in KO mice than in WT mice (*p* = 0.033, Supplementary Figure 1). MRI on day 7 revealed cerebral infarction in the KO mouse that died on day 11 (Fig. 1), but not in any of the EC-Tg or WT mice.

In the present study, CBF data obtained by MRI before the death of the mice were included in the analysis, whereas evaluation of angiogenesis and histological analysis of the three KO and one WT mice that died were excluded as we could not conduct immediate autopsies that would have minimized the effects of hypoxic changes in the brain.

### Temporal Profiles of Regional CBF Recorded by ASL

**Figure 2:**
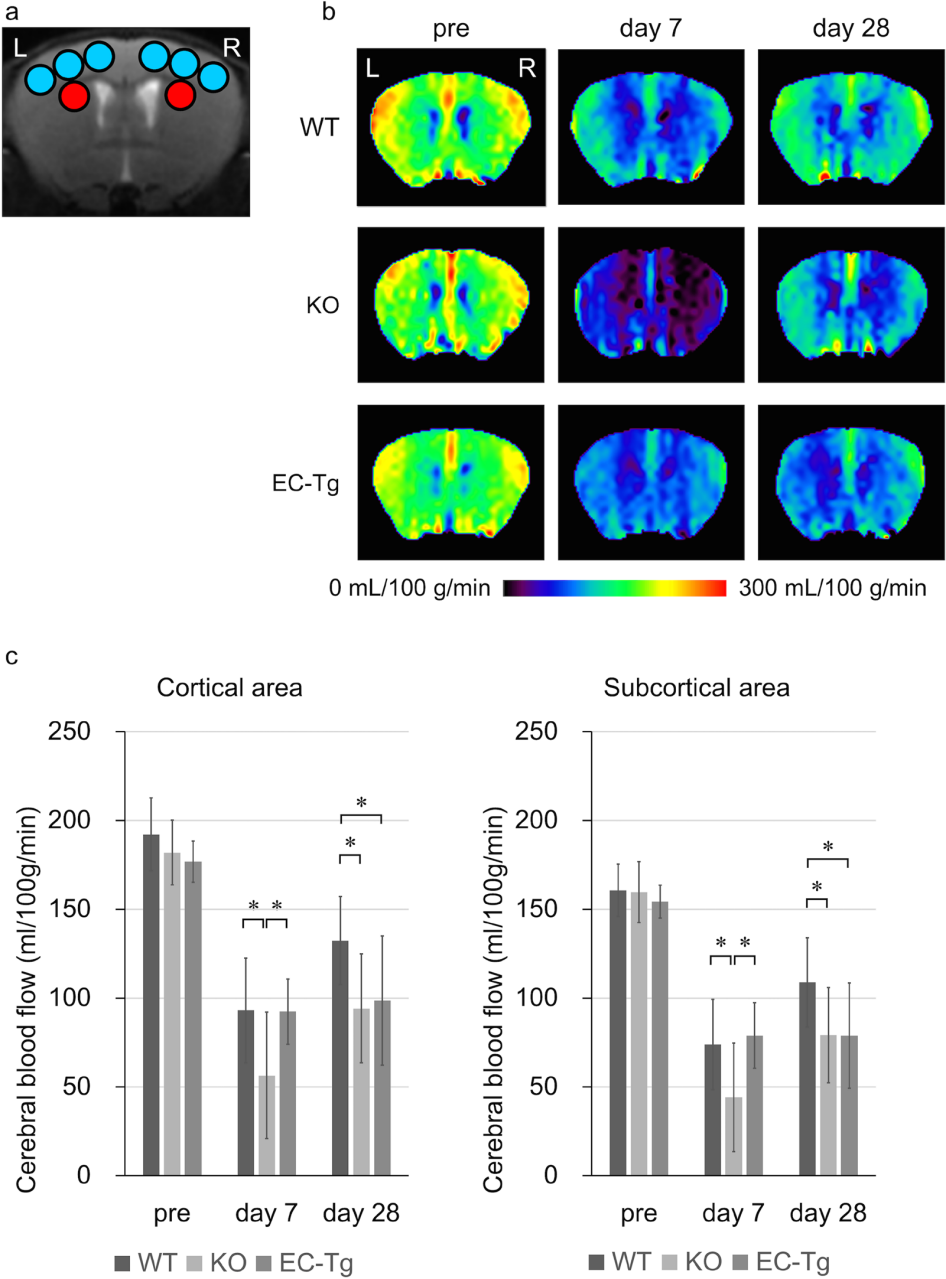
Temporal profiles of cerebral blood flow (CBF) of mice with bilateral common carotid artery stenosis (BCAS). (**a**) Regions of interest (ROIs) used in the analyses of CBF images obtained from arterial spin-labelling magnetic resonance imaging. The CBF values in the cerebral cortex and the subcortical area were calculated from the six blue and two red ROIs, respectively. (**b**) Representative coronal CBF images obtained from arterial spin labelling in WT, KO and EC-Tg mice. (**c**) Temporal profiles of CBF presented as absolute values (mL/100 g/min) in the cortical and subcortical parenchymal areas. A column with a bar represents mean ± SD of CBF. Two-way repeated measures ANOVA was conducted for CBF values between genotypes and time interaction terms. In the cortical area, genotypes significantly affected the CBF values (*p* = 0.012) and the interaction was marginally significant (*p* = 0.066). In the subcortical area, both genotypes (*p* = 0.049) and interaction (*p* = 0.014) were significant. Additionally, in both the cortical and the subcortical areas, there were significant differences in the CBF values between the three genotypes using one-way ANOVA on day 7 (Cortical, *p* = 0.045; Subcortical, *p* = 0.046 (WT, *n = *15; KO, *n = *7; EC-Tg, *n = *8)) and day 28 (Cortical, *p* = 0.035; Subcortical, *p* = 0.048 (WT, *n = *14; KO, *n* = 5; EC-Tg, *n = *8), but not pre-surgery (pre) (Cortical, *p* = 0.17; Subcortical, *p* = 0.61 (WT, *n* = 15; KO, *n* = 8; EC-Tg, *n* = 8)). On day 7, CBF in KO mice was significantly decreased compared with the other genotypes according to Tukey’s test. On day 28, CBF in KO and EC-Tg mice was significantly decreased compared with WT mice; *p < 0.05.

We evaluated CBF values in the cortical and subcortical areas before and after BCAS surgery (Fig. 2). The CBF values in the cortical and subcortical areas were calculated as the mean value of CBF in the blue and red regions of interest (ROIs), respectively (Fig. 2a).

In the cortical area, there was no significant difference between genotypes before surgery (CBF values (ml/100 g/min) in WT (*n* = 15), 192 ± 21; KO (*n* = 8), 182 ± 18; and EC-Tg (*n* = 8), 177 ± 12; *p* = 0.17; one-way ANOVA) (Fig. 2b,c). However, as shown in Fig. 2c, on day 7 after BCAS surgery, the CBF in KO mice (*n* = 7, excluding one mouse which died before day 7) decreased markedly to 29.7%, which was significantly lower than that of the other genotypes (49.3% in WT mice, *p* = 0.038; 52.8% in EC-Tg mice, *p = *0.019; Tukey’s test). On day 28, CBF in WT mice (*n* = 14, excluding one mouse that died during MRI) was restored to 69.5% of the value before surgery, whereas values in KO mice (*n* = 5, excluding two further mice that died from day 7 to day 28; 50.0%, *p* = 0.031; Tukey’s test) and EC-Tg mice (56.1%, *p* = 0.037; Tukey’s test) were significantly lower than in WT mice (Fig. 2b,c). Additionally, two-way repeated measures ANOVA, which was conducted for CBF values between genotypes and time interaction terms, showed that genotypes (*p* = 0.012) and time (*p* < 0.0001) significantly affected the CBF values, and that the interaction was marginally significant (*p* = 0.066).

The temporal changes of CBF in the subcortical area also showed a similar tendency as the cortical area (Fig. 2b,c). No significant differences were observed between the three genotypes before surgery (CBF values (ml/100 g/min) in WT (*n* = 15), 161 ± 15; KO (*n* = 8), 160 ± 17; EC-Tg (*n* = 8), 154 ± 9; *p* = 0.61; one-way ANOVA). On day 7, CBF values in WT, KO (*n = *7, excluding one mouse which died before day 7) and EC-Tg mice decreased to 46.6%, 26.6% and 51.4%, respectively compared with their baseline levels, with the CBF in KO mice being significantly decreased compared with the other mice (KO vs WT, *p* = 0.029; KO vs EC-Tg, *p = *0.024; Tukey’s test). On day 28, CBF values in WT (*n* = 14, excluding one dead mouse during MRI), KO (*n* = 5, excluding further two mice which died from day 7 to day 28) and EC-Tg mice were 68.2%, 47.7%, and 51.3% of their respective baseline levels, with the levels in KO and EC-Tg mice being significantly decreased compared with WT mice (*p* = 0.031 and 0.047, respectively; Tukey’s test). By two-way ANOVA, all three factors were significant (Genotypes, *p* = 0.049; Time, *p* < 0.0001; Interaction, *p* = 0.014). Collectively, these data indicate that the early adaption of CBF to surgery was impaired in KO mice, and that late adaption of CBF was impaired in both KO and EC-Tg mice.

To speculate on the probable causes of death of the mice, the temporal changes of CBF in these mice were evaluated and are presented in Supplementary Table 1. The CBF in one dead KO mouse (Animal Code: KO-3 in Supplementary Table 1) was prominently decreased the day before death (cortical, 2.9 ml/100 g/min; subcortical, 2.2). Another KO mouse (KO-5) that died also had a severe reduction in CBF, although not to the same extent as KO-3, on day 7 (cortical, 62.7 ml/100 g/min; subcortical, 47.5), and additionally had cerebral infarction as determined by MRI. These results suggest that death in the KO mice was triggered by severe cerebral hypoperfusion that led to cerebral infarction. However, in the WT mouse that died during anaesthesia (WT-7) the reduction in CBF (cortical, 98.5 ml/100 g/min; subcortical, 78.4) was comparable to those of the other WT mice.

### Brain MRA

**Figure 3:**
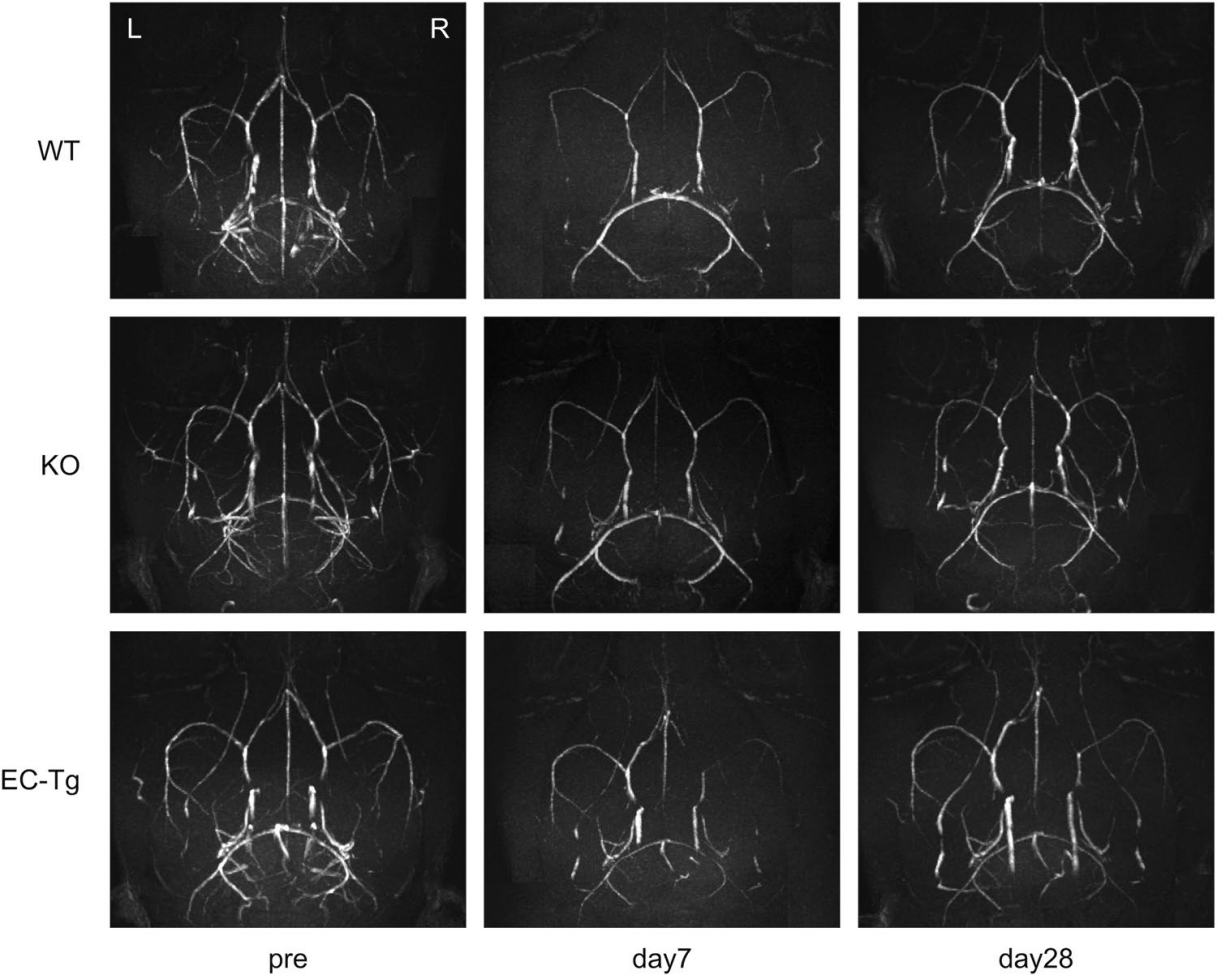
Intracranial arterial flow after BCAS assessed by magnetic resonance angiography (MRA). Representative images of intracranial arterial flow in WT, KO, EC-Tg mice are shown. Images were obtained by a 7 Tesla brain MRA before (pre) and at 7 and 28 days after BCAS.

The development of collateral arteries, such as the posterior communicating artery, showed no differences between genotypes prior to surgery. In mice of all genotypes, MR angiography (MRA) showed that the signal in the anterior circulation was prominently decreased on day 7 after surgery and that incomplete recovery of the signal was observed on day 28. No obvious vascular stenosis/occlusion or moyamoya vessels were observed in mice of any genotype (Fig. 3).

### Histopathological analysis for Glut1 and Klüver-Barrera staining

**Figure 4:**
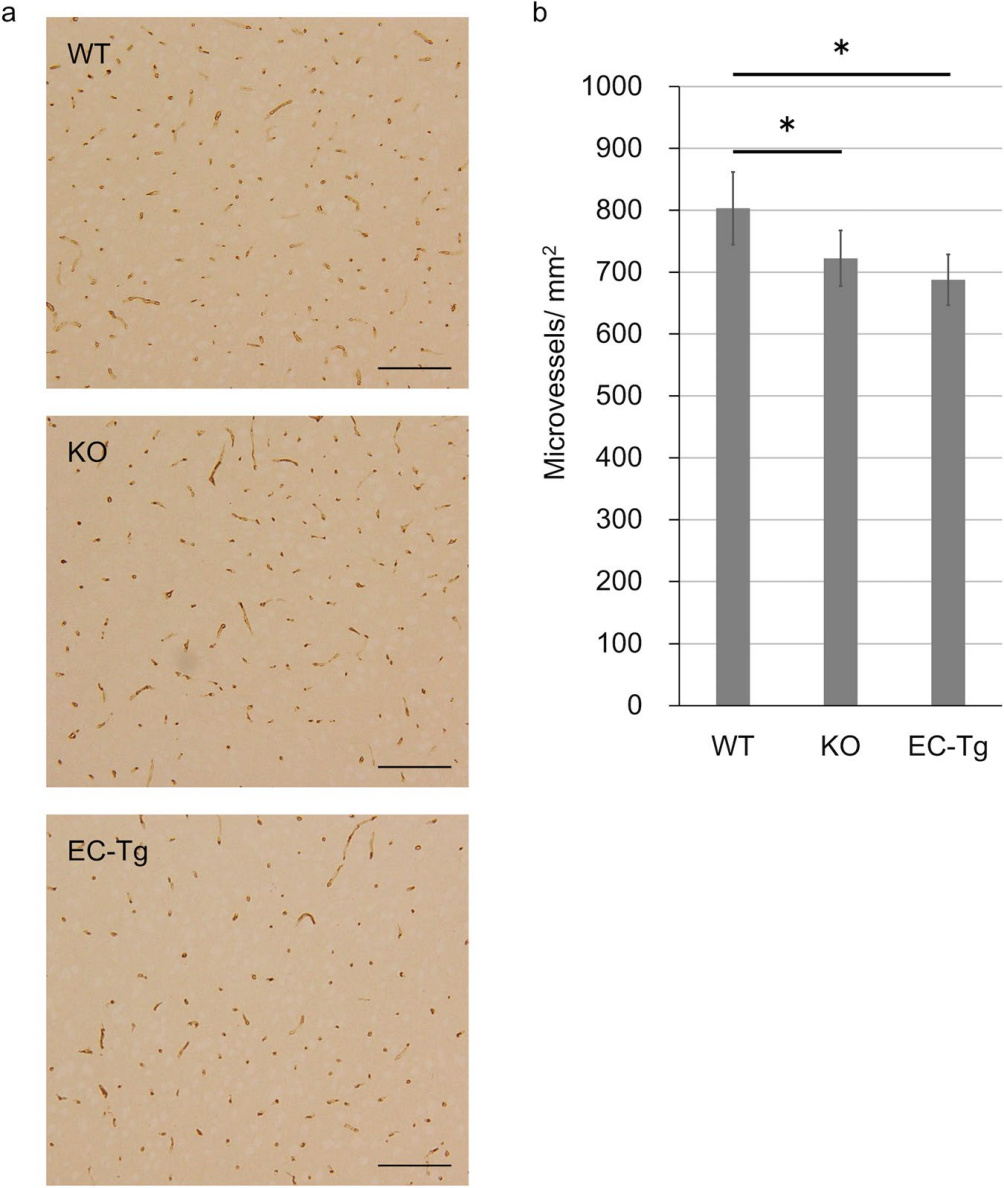
Cerebral cortex stained for glucose transporter 1 (Glut1) of surviving mice. (**a**) Representative images of Glut1-immunostained sections of cerebral cortex of WT, KO and EC-Tg mice at 28 days after BCAS. Scale bar represents 100 μm. (**b**) Quantified results of cerebral microvessels. A column with a bar represents mean ± SD of the number of cerebral microvessels/mm^2^. There was a significant difference in the number of cerebral microvessels between the three genotypes using one-way ANOVA (WT, *n* = 6; KO, *n* = 5; EC-Tg, *n* = 8; *p = *0.002). There were significant differences between KO and WT (*p* = 0.034) and between EC-Tg and WT (*p* = 0.001) using Tukey’s test; *p < 0.05 vs WT.

We evaluated the extent of cerebral angiogenesis by glucose transporter 1 (Glut1) immunohistochemistry on day 28 after BCAS in the surviving KO (n = 5) and EC-Tg (n = 8) mice, and in WT mice (n = 6) randomly-selected from the 14 surviving WT mice, where one mouse, which died during anaesthesia, was excluded (Fig. 4). The density of cerebral microvessels (number per mm^2^) in KO and EC-Tg mice was significantly lower than in WT mice (KO vs WT, *p* = 0.034; EC-Tg vs WT, *p* = 0.001), suggesting that angiogenesis was suppressed in KO and EC-Tg mice under chronic oligaemic conditions. Additionally, we evaluated white matter lesions by Klüver-Barrera staining, but no differences between genotypes were observed (Supplementary Figure 2).

## Discussion

*RNF213* was identified as a susceptibility gene for MMD^1,2^ and was also shown to be significantly associated with intracranial arterial stenosis^2,4^. However, obvious vascular abnormalities have not been observed in *Rnf213* genetically modified mouse models^12–15^, even though inhibition of *rnf213* expression in zebrafish was found to induce abnormal and sprouting vessels in the craniocervical region^2^. Here, we have now demonstrated marked phenotypic changes, such as the reduction in CBF, in mice under cerebral hypoperfusion.

In this study, we first subjected *Rnf213* KO and EC-Tg mice to BCAS surgery, which is known to induce chronic cerebral hypoperfusion and endothelial dysfunction^22–25^, and then estimated their CBF levels at the early (day 7) and late (day 28) postoperative stages. We found that KO mice displayed a more severe and significant reduction in CBF at the early postoperative stage than WT mice. These reduced CBF levels resulted in cerebral infarction in the KO mice, but not in any of the WT mice in the BCAS model^26^. Furthermore, 37.5% (three out of eight) of KO mice died after BCAS surgery, whereas all WT mice survived chronic hypoperfusion. Restoration of CBF was also impaired in the KO mice on day 28 after BCAS. It is of interest that EC-Tg mice could adapt to BCAS at the early stage after surgery, but CBF recovery was low, and equivalent to that of KO mice, on day 28 after BCAS. These observations suggest that *RNF213* plays an important role in the maintenance of CBF.

It is well known that cerebral hypoperfusion is compensated by two different mechanisms, arteriogenesis and angiogenesis^20,21^. Under cerebral hypoperfusion, arteriogenesis refers to the expansion and remodelling of pre-existing arterioles and is triggered by an increase in fluid shear stress, while angiogenesis is defined as the growth and proliferation of new vessels from existing vasculature and is induced by hypoxia^20,21^. Whereas the former mechanism functions in the early phase, such as on day 7 after BCAS in mice, the latter process works in later phases, such as after 14 days after BCAS^27^. In the BCAS model, both mechanisms appear to compensate for cerebral hypoperfusion^25,28^.

At the early stage, KO mice but not EC-Tg mice showed a significant reduction in CBF compared with WT mice, and this could be due to a lower extent of arteriogenesis. Indeed, *RNF213* has been reported to influence arteriogenesis^12,14^. In the common carotid artery ligation model, thickening of the intima and media, which are histological characteristics of arteriogenesis^29^, was not observed in KO mice^12^. Additionally, expression of matrix metalloproteinase-9 (MMP-9), which is involved in vascular remodelling, was decreased in the endothelium of KO mice^30^. In contrast, *Rnf213* p.R4757K knock-in mice did not show such phenomena^14^. These findings suggest that arteriogenesis could be impaired in KO mice under oligaemic conditions due to suppressed vascular remodelling.

Conversely, at the late stage, CBF levels were significantly lower in KO and EC-Tg mice than WT mice, suggesting that CBF restoration was impaired in both KO and EC-Tg mice. Histopathological analysis further demonstrated that angiogenesis in the cerebral cortex was impaired in both EC-Tg and KO mice. This reduced angiogenesis is therefore considered as one of the primary causes of poor CBF improvement at the late stage in both models. Previously, we reported that angiogenesis was impaired in EC-Tg mice but not in KO mice under hypoxic conditions^13^. Although there was some discordance between the effects of hypoperfusion and hypoxia on angiogenesis in KO mice, the findings were consistent under both conditions for EC-Tg mice. This inconsistency might be explained by the effects of haemodynamic factors, which is a major distinction between hypoperfusion and hypoxia. Further investigation is required to clarify the underlying mechanisms.

Although the exact molecular mechanisms by which *RNF213* regulates angiogenesis and arteriogenesis remain largely unknown, previous studies have suggested several possible processes. In regard to angiogenesis, *RNF213* may be involved in two different signalling pathways. The first is a process mediated by the hypoxia-inducible factor-1 (HIF-1), which is considered as a master regulator of angiogenesis^31^. Banh *et al*. reported that in Her2+ breast cancer cells under hypoxic conditions, *RNF213* serves as a substrate of protein-tyrosine phophatase-1B and affects HIF-1 by regulating α-ketoglutarate-dependent dioxygenases^32^. In normoxic conditions, HIF-1α is degraded by the 26 s proteasome but, under hypoxic conditions, HIF-1α is stabilised, binds with HIF-1β, and activates transcription of several genes that are essential for angiogenesis, such as those encoding vascular endothelial growth factor, placental growth factor, and stromal cell-derived factor 1^31^. Therefore, *RNF213* dysfunction could affect regulation of the HIF-1 pathway. A second possible signalling pathway may function through caveolin-1, the principal structural component of caveolae. Caveolin-1 is expressed by different vascular cells and is involved in the maintenance of vascular homeostasis, including vesicular trafficking and signal transduction^33,34^. Serum caveolin-1 levels were found to be decreased in MMD patients and were further decreased in those carrying the *RNF213* p.R4810K variant^35^. Cerebral angiogenesis was also decreased under cerebral ischemia in caveolin-1 knockout mice^36^, suggesting that RNF213 may affect angiogenesis via caveolin-1.

However, regarding arteriogenesis, *RNF213* could affect immune cells, including monocytes, helper T cells, and regulatory T cells that play crucial roles in regulating vascular remodelling^20^. Arteriogenesis is triggered by fluid shear stress. Increased shear stress induces endothelial activation, with subsequent upregulation of cell adhesion molecules and chemokines available for circulating leukocytes, which then promote vascular remodelling^20^. As *RNF213* is predominantly expressed in human immune tissues, such as spleen, leukocytes, and lymph nodes, involvement of *RNF213* in the immune system has been proposed^1^. Kanoke *et al*. reported that the ratio of regulatory T cells in KO mice after the administration of immunological adjuvant was significantly decreased, and proposed that *RNF213* could play a role in the differentiation of regulatory T cells^15^. Regulatory T cells play pivotal roles in the maintenance of immunological self-tolerance and immune homeostasis^37^. These findings suggest that *RNF213* dysfunction may affect vascular remodelling due to abnormalities in the immune system.

This study has intriguingly demonstrated that ablation of *RNF213* suppresses both arteriogenesis and angiogenesis, whereas expression of the EC-specific *RNF213* p.R4810K variant inhibits only angiogenesis but not arteriogenesis. At present, there is no clear explanation as to why arteriogenesis is inhibited in the KO mice but not in the EC-specific RNF213 p.R4810K variant. While further study is essential to explain this difference, it is of interest to note that a reduced angiogenesis phenotype was found *in vivo* in both KO and EC-Tg mice, and consistently confirmed by several independent groups using different biological specimens, such as iPS-derived ECs or endothelial progenitor cells from patients with MMD^17,38,39^, as well as cultured ECs overexpressing different *RNF213* variants found in Asian or Caucasian patients^18^. A reduced CBF, which is one of the characteristics of MMD patients^8^, is also manifest in both KO and EC-Tg mice. These findings collectively suggest that a common molecular mechanism may function as a result of both KO and *RNF213* p.R4810K overexpression; one in which *RNF213* p.R4810K reduces CBF levels by lowering angiogenesis. Clearly, further experiments are required to understand the molecular processes by which RNF213 and p.R4810K modulate CBF levels through angiogenesis in hypoxia and hypoperfusion.

This study has one limitation. As only KO mice died at a high rate after BCAS, survivor bias may be present. In particular, the CBF values and pathological evaluation at day 28 after BCAS may be underestimated in the KO mice, as animals that died following the procedure were more likely to have had greater impairments in CBF that could not have been examined. Nevertheless, we were able to conclude that CBF was reduced in the KO mice.

In conclusion, the present study suggests that *RNF213* plays an important role in CBF maintenance under ischemic conditions by affecting angiogenesis and arteriogenesis. In addition, a chronic hypoperfusion model such as BCAS could be a promising model for investigating the role of *RNF213* in steno-occlusive diseases, including MMD. Further animal and molecular studies are needed to reveal the underlying mechanisms by which *RNF213* affects the vascular system.

## Methods

### Experimental animals

In this study, we used 8–10-week-old KO, EC-Tg and WT mice. All mice used were on the C57BL/6 background. The generation of KO and EC-Tg mice were as previously described^11,13^. Briefly, KO mice were generated via targeted disruption of exon 20 of *Rnf213* with the Cre/loxP system. Heterozygous male and female mice were intercrossed to produce homozygous offspring. Genotyping was performed by the polymerase chain reaction (PCR) using the primers described previously^11^. The Tg construct consisted of the loxP-flanked transcription termination sequence (beta globin poly(A)) placed between the cytomegalovirus enhancer fused to the chicken beta-actin promoter (CAG promoter) and the mouse *Rnf213* p.R4757K mutant coding sequence. Genotypes of Tg offspring were determined by PCR using the primers described previously^13^. To obtain mice harbouring vascular ECs overexpressing *Rnf213*, Tg founders were bred with mice expressing the Cre transgene under direction of the Tie2 promoter/enhancer, which is expressed in endothelial cells^13^.

All animal experiments and animal care protocols were in accord with the Animal Welfare Guidelines of Kyoto University (Kyoto, Japan) and the National Cerebral and Cardiovascular Center (Osaka, Japan). The experimental protocol was authorized by the Internal Animal Welfare Committee at Kyoto University (approval no. Med Kyo17051; approval date: 27/3/2017) and the National Cerebral and Cardiovascular Center (approval no. 15024; approval date: 1/4/2015). All procedures were performed under anaesthesia and all efforts were made to minimise suffering.

### Study design

BCAS surgery was conducted in three groups of 8–10-week-old mice: (1) KO (*n* = 8), (2) EC-Tg (*n* = 8), and (3) WT mice (*n* = 15). CBF was measured with ASL and with brain MRA (7 Tesla, BioSpec 70/30 USR; Bruker BioSpin, Ettlingen, Germany) before, and 7 days and 28 days after BCAS surgery. MRA was also used for evaluating vascular morphology. Additionally, histological analysis of the brains was performed after MR scanning on day 28.

### Surgical Procedure for BCAS Surgery

BCAS is known to be a model for vascular cognitive impairment^22,24^. The severity of cerebral hypoperfusion can be modified by altering the diameter of the coils and, in most studies, 0.18 mm coils have been used. The 0.18 mm coils can lead to cerebral hypoperfusion over 3 months without affecting blood pressure^22–25^.

The BCAS procedure was as described previously^22^. Anaesthesia was induced with 2% isoflurane and maintained with 1.5% isoflurane in 80% nitrous oxide and 20% oxygen. Rectal temperature was maintained between 36.5 °C and 37.5 °C. Through a midline cervical incision, both common carotid arteries were exposed. Microcoils with an internal diameter of 0.18 mm (Sawane Spring, Hamamatsu, Japan) were applied to the bilateral common carotid arteries.

### MR Imaging/MRA and ASL Parameters

All MR scans were performed using a 7 Tesla horizontal bore imaging system equipped with a gradient system capable of a maximum gradient amplitude of 669 mT/m and a slew rate of 7989 T/m/s. Radiofrequency transmission was performed using an 86 mm inner diameter volume coil. Signals were detected using a four-channel receive-only phased-array surface coil. The mice were anaesthetised using isoflurane (4% for induction and 1.0–2.5% for maintenance) in 1.2 L/min room air mixed with 0.1 L/min oxygen. Each animal was placed in a prone position, and the head fixed with a bite bar and ear bars. Body temperature was monitored by a rectal thermometer and maintained with a warm waterbed and warm air. Heart rate and respiratory rate were continuously monitored. T2-weighted images were acquired using a rapid acquisition with a relaxation enhancement (RARE) sequence with the following parameters: RARE factor, 8; TR/TE, 2500/35.14 ms; number of averages, 2; matrix size, 200 × 200; FOV, 2.0 × 2.0 cm^2^; in-plane spatial resolution, 100 × 100 μm^2^; slice thickness, 1.0 mm, gapless; number of slices, 20; and scan time, 2 min 5 s. Diffusion weighted images were acquired using a spin-echo echo-planar imaging sequence with the following parameters: two shots; TR/TE, 5,000/28.38 ms; number of averages, 1; matrix size, 100 × 100; FOV, 2.0 × 2.0 cm^2^; in-plane spatial resolution, 200 × 200 μm^2^; slice thickness, 1.0 mm, gapless; number of slices, 20; b-value, 1000 s/mm^2^, 30 directions; and scan time, 5 min 50 s. In diffusion weighted images, a generalised, autocalibrating, partially parallel acquisition technique was used with an acceleration factor of 2. Apparent diffusion coefficient (ADC) maps were calculated from the diffusion-weighted images. The 3D time-of-flight (TOF) MRA images were acquired using a fast low-angle shot sequence with the following parameters: TR/TE, 10.19/3.54 ms; number of averages, 3; matrix size, 280 × 280 × 170; FOV, 1.68 × 1.68 × 1.02 cm^3^; spatial resolution, 60 × 60 × 60 μm^3^; and scan time, 18 min 12 s. In 3D TOF MRA, tilted optimised nonsaturating excitation pulse and flow compensation were used. Maximum intensity projection images were reconstructed using Osirix software (Pixmeo, Switzerland).

CBF measurement of coronal slices was carried out using a flow-sensitive alternating inversion recovery technique^40,41^, an ASL-based method. In each of the non-selective and slice-selective experiments, twenty-two images with different inversion times were acquired using a RARE sequence with the following parameters: RARE factor, 72; TR/TE, 10000/46 ms; number of averages, 1; matrix size, 128 × 128; FOV, 4.0 × 4.0 cm^2^; in-plane spatial resolution, 313 × 313 µm^2^; slice thickness, 1.0 mm; and number of slices, 1. The following inversion time values were used: 30, 100, 200, 300, 400, 500, 600, 700, 800, 900, 1000, 1100, 1200, 1300, 1400, 1500, 1600, 1700, 1800, 1950, 2100, and 2300 msec. Total scan time was 8 min 24 s. The CBF image was calculated from the obtained 44 images using ParaVison 5.1 (Bruker BioSpin). Absolute CBF values were calculated from the T1 relaxation time difference between nonselective and slice-selective experiments. The CBF images were acquired at bregma level. ROI analyses of CBF images were carried out using the Dr. View/LINUX R2.5.0 program (Asahi Kasei Information System, Tokyo). The ASL images were co-registered to the T2-weighted images for selection of ROIs by using the Dr. View/LINUX. In the corresponding slices of the T2-weighted image, circular ROIs with a diameter of 1 mm were symmetrically placed on the cerebral cortex region and subcortical regions, including the corpus callosum, caudoputamen and hippocampus, and superimposed on CBF images.

### Histological analysis of brains

At 28 days after BCAS, mice were anaesthetised and perfused with PBS with 1 U/mL of heparin. Brains were removed, fixed in 10% formaldehyde, embedded in paraffin, and sectioned. We selected all surviving KO (*n* = 5) and EC-Tg (*n* = 8) mice and randomly selected six out of fourteen surviving WT mice (excluding one mouse which died during anaesthesia because the brain was not properly removed due to unexpected death). Sections were stained with mouse anti-Glut1 antibody for evaluating cerebral angiogenesis^13,42^ and by Klüver-Barrera staining for evaluating demyelinating changes of white matter lesions^43^. Histological images were obtained using an Olympus BX43F connected to an Olympus DP21 digital camera. We counted Glut1-positive capillaries in the cerebral cortex from three sections per mouse using Image J software (version 1.50f3; National Institutes of Health, USA). White matter lesions were graded as normal (grade 0), disarrangement of nerve fibres (grade 1), formation of marked vacuoles (grade 2), and disappearance of myelinated fibres (grade 3), as previously described^43^.

### Statistical analysis

Data are presented as mean ± SD unless otherwise indicated. Survival duration was calculated from the date of BCAS surgery, and survival was defined as survival until 28 days after the surgery. The Kaplan–Meier method was used to estimate survival, and differences in survival were assessed by the log-rank test with a Bonferroni correction. CBF values were compared by two-way repeated measures ANOVA and one-way ANOVA with Tukey’s *post hoc* test. Other statistical analyses were carried out by one-way ANOVA with Tukey’s *post hoc* test. A *p* value < 0.05 was considered statistically significant, and a *p* value of < 0.1 and ≥ 0.05 was considered marginally significant. All data analyses were carried out using JMP pro version 11.2.0 (SAS Institute, Cary, NC).

## Electronic supplementary material

Dataset 1
