# Supplementary material for: Dysregulation of RNF213 promotes cerebral hypoperfusion
Source: Sci Rep. 2018 Feb 26;8:3607. doi: 10.1038/s41598-018-22064-8 (PMC5827518; doi:10.1038/s41598-018-22064-8)
Supplement: Supplementary file 1 — Dataset 1 [file 41598_2018_22064_MOESM1_ESM.doc]

**Supplementary Information**

**Dysregulation of *RNF213* promotes cerebral hypoperfusion**

Takaaki Morimoto, Jun-ichiro Enmi, Yorito Hattori, Satoshi Iguchi, Satoshi Saito, Kouji H. Harada, Hiroko Okuda, Yohei Mineharu, Yasushi Takagi, Shohab Youssefian, Hidehiro Iida, Susumu Miyamoto, Masafumi Ihara, Hatasu Kobayashi*, Akio Koizumi

Correspondence to Hatasu Kobayashi (hatasu-kobayashi@isc.chubu.ac.jp).

| Animal  Code | Cortical CBF | |  | Subcortical CBF | | Comments |
| --- | --- | --- | --- | --- | --- | --- |
|  | pre | day 7 |  | pre | day 7 |  |
| KO-3 | 148.7 | 2.9 |  | 137.5 | 2.2 | Died on day 8 |
| KO-5 | 189.4 | 62.7 |  | 161.4 | 47.5 | Died on day 11  Cerebral infarction (detected by MRI on day 7) |
| KO-8 | 183.5 | NA |  | 158.8 | NA | Died on day 1 |
| WT-7 | 174.9 | 98.5 |  | 154.1 | 78.4 | Accidentally died during aesthesia for MRI on day 28 |

**Supplementary Table 1.** Quantitative values of CBF (mL/100 g/min) of dead mice are presented. CBF, cerebral blood flow; KO, *Rnf213* knockout; WT, wild type.

Time (day)

KO

WT

EC-Tg

*

Survival rate

**Supplementary Figure 1.** Kaplan-Meier survival curve of KO (*n* = 8), EC-Tg (*n* = 8) and WT mice (*n* = 15) after BCAS surgery. There is a significant difference between KO mice and WT mice (*p* = 0.033, log-rank test with Bonferroni correction); * the curves of WT and EC-Tg mice overlap.


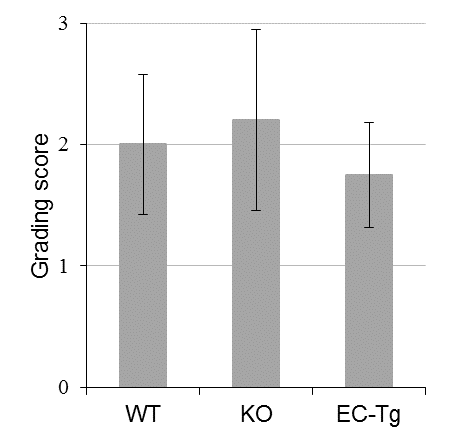

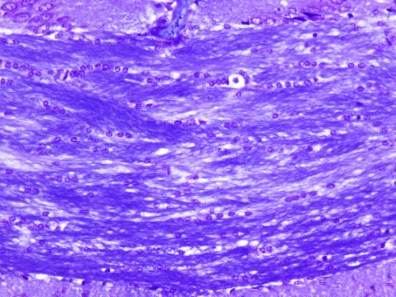

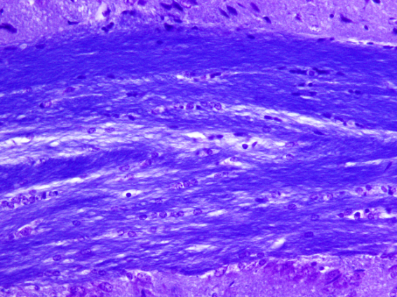

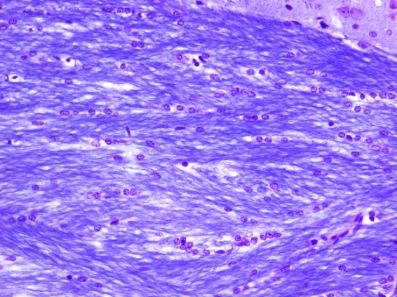


KO

WT

EC-Tg

a

b

**Supplementary Figure 2.** Corpus callosum with Klüver-Barrera staining.

(A) Representative photomicrographs from mice of each genotype. Scale bars represent 50 μm.

(B) The grading scores of white matter lesions. White matter lesions were graded as normal (grade 0), disarrangement of nerve fibres (grade 1), formation of marked vacuoles (grade 2), and disappearance of myelinated fibres (grade 3), as previously described^1^. Values are expressed as mean ± SD. No significant difference was observed between the three genotypes.

**Reference**

1. Wakita, H., Tomimoto, H., Akiguchi, I. & Kimura, J. Protective Effect of Cyclosporin A on White Matter Changes in the Rat Brain After Chronic Cerebral Hypoperfusion. *Stroke* **26,** (1995).
